# Management of Non-Metastatic Non-Small Cell Lung Cancer (NSCLC) with Driver Gene Alterations: An Evolving Scenario

**DOI:** 10.3390/curroncol31090379

**Published:** 2024-08-30

**Authors:** Valeria Fuorivia, Ilaria Attili, Carla Corvaja, Riccardo Asnaghi, Ambra Carnevale Schianca, Pamela Trillo Aliaga, Ester Del Signore, Gianluca Spitaleri, Antonio Passaro, Filippo de Marinis

**Affiliations:** 1 Division of Thoracic Oncology, European Institute of Oncology IRCCS, 20141 Milan, Italy; 2 Department of Oncology and Hemato-Oncology, University of Milan, 20141 Milan, Italyantonio.passaro@ieo.it (A.P.);

**Keywords:** tyrosine kinase inhibitors (TKI), adjuvant, neoadjuvant, perioperative, locally advanced, early stage, driver gene, targeted treatments, NSCLC

## Abstract

The ever-growing knowledge regarding NSCLC molecular biology has brought innovative therapies into clinical practice; however, the treatment situation in the non-metastatic setting is rapidly evolving. Indeed, immunotherapy-based perioperative treatments are currently considered the standard of care for patients with resectable NSCLC in the absence of *EGFR* mutations or *ALK* gene rearrangements. Recently, data have been presented on the use of tyrosine kinase inhibitors (TKIs) in the adjuvant and locally advanced setting for patients with NSCLC harboring such driver gene alterations. The aim of the current work is to review the available evidence on the use of targeted treatments in the non-metastatic setting, together with a summary of the ongoing trials designed for actionable gene alterations other than *EGFR* and *ALK*. To date, 3-year adjuvant osimertinib treatment has been demonstrated to improve DFS and OS and to reduce CNS recurrence in resected *EGFR*-mutated NSCLC in stage IB–IIIA (TNM 7th edition). The use of osimertinib after chemo-radiation in stage III unresectable *EGFR*-mutated NSCLC showed the relevant PFS improvement. In the *ALK*-positive setting, 2-year alectinib treatment was shown to clearly improve DFS compared to adjuvant standard chemotherapy in resected NSCLC with stage IB (≥4 cm)–IIIA (TNM 7th edition). Several trials are ongoing to establish the optimal adjuvant TKI treatment duration, as well as neoadjuvant TKI strategies in *EGFR-* and *ALK*-positive disease, and (neo)adjuvant targeted treatments in patients with actionable gene alterations other than *EGFR* or *ALK*. In conclusion, our review depicts how the current treatment scenario is expected to rapidly change in the context of non-metastatic NSCLC with actionable gene alterations, hence appropriate molecular testing from the early stages has become crucial to establish the most adequate approaches both in the perioperative and the locally advanced disease.

## 1. Introduction

Non-small-cell lung cancer (NSCLC) is the leading cause of cancer-related death worldwide, and only approximately 25% of patients are diagnosed with it at an early stage and are thus amenable to radical surgery [1,2,3]. On the other hand, locally advanced disease accounts for almost one-third of NSCLC cases, presenting a wide range of clinical and pathological heterogeneity, including potentially resectable and unresectable stage III disease [2].

Despite radical surgery, disease recurrence occurs in more than half of the patients, ranging from ~60% in resected stage I to ~80% in resected stage III disease at 5 years [4]. Adjuvant or neoadjuvant cisplatin-based chemotherapy has been the one-size-fits-all standard of care for patients with radically resected stage II–III NSCLC [1,5,6,7,8,9,10,11].

Notably, the historical survival rates (ranging from 60% in N0 to 20% in N2 disease) [1,2] have overall markedly improved with the adoption of immunotherapy-based regimens, both in the adjuvant, perioperative [12,13,14,15,16], and consolidation settings after chemo-radiation [17], as well as in the metastatic setting in patients without oncogene-addicted disease.

Finally, unresectable locally advanced NSCLC refers to stage IIIB (with exception of T3N2 that are deemed to be potentially resectable) and IIIC disease, according to the eighth edition of the Tumor Node Metastases (TNM) classification, but the definition is also extended to stage II cases that are deemed not resectable due to technical or functional reasons [12,18]. At the time of the current review, the standard of care for this heterogeneous group of patients is platinum-based chemotherapy with concurrent radiation therapy (RT), followed by consolidation treatment with durvalumab for one year, still with no formal need for molecular testing [12,19].

Conversely, driver actionable gene alterations have been widely explored in the metastatic setting with favorable results, and several targeted treatment options are available as front-line treatments or in the pretreated setting [20,21,22,23,24,25]. Overall, approximately 50–70% Asian and 30–40% non-Asian patients with metastatic non-squamous NSCLC carry druggable mutations (epidermal growth factor receptor *EGFR*, anaplastic lymphoma kinase *ALK*, ROS proto-oncogene 1 *ROS1*, rearranged during transfection *RET*, B-Raf proto-oncogene *BRAF*, MET proto-oncogene *MET*, Kirsten rat sarcoma virus *KRAS*, Neurotrophic tyrosine receptor kinase *NTRK*), and the use of the appropriate targeted drugs results in a manifold increase in their overall survival [21,26].

Given the remarkable outcomes obtained with targeted therapies in patients with advanced NSCLC harboring oncogenic drivers, these agents are being investigated in the non-metastatic setting, including resectable stage I–III and unresectable stage III disease, with perioperative (neoadjuvant and/or adjuvant) and consolidation strategies [27,28].

To date, following the positive disease-free survival (DFS) and overall survival (OS) results of the ADAURA trial, adjuvant osimertinib for 3 years has become the standard treatment option in patients with stage IB–III resected NSCLC with *EGFR* common activating mutations (exon 19 deletions or exon 21 p.L858R point mutation), after optional adjuvant chemotherapy [29]. In parallel, positive DFS results from the ALINA trial led to the approval of alectinib by the Food and Drug Administration (FDA) and European Medicines Agency (EMA) for use in the adjuvant setting of patients with stage IB (≥4 cm)–IIIA (TNM 7) resected NSCLC with *ALK* gene rearrangements [30]. Very recently, positive DFS results were presented on osimertinib consolidation vs. placebo after chemo-radiation in an unresectable *EGFR*-mutant NSCLC in the phase III LAURA trial [31].

Overall, these results demonstrate how expanding TKI treatments to earlier settings might improve clinical outcomes, offering new therapeutic strategies that alter the current standard of care and confirming the absence of any role for immune checkpoint inhibitors (ICIs) in oncogene-addicted tumors, at least in those *EGFR-* and *ALK*-positive ones and in the non-metastatic stages.

This review aims to shed light on the available evidence on targeted treatments in non-metastatic settings in patients diagnosed with NSCLC harboring driver genetic alterations, while depicting the future potential evolution of treatment strategies based on recent data and ongoing clinical trials.

## 2. *EGFR*-Mutant Setting

### 2.1. Adjuvant Targeted Treatments in EGFR-Mutant Setting

Approximately 10–20% and 50% of NSCLC patients in the Caucasian and Asian population, respectively, harbor *EGFR* activating mutations [32].

First-generation *EGFR*-TKIs, gefitinib, erlotinib, and icotinib, have shown superior response rates and longer survival compared to chemotherapy in advanced NSCLC, leading to studies on their adjuvant use in resected NSCLC. At first, retrospective and cohort studies indicated DFS improvements with adjuvant gefitinib and erlotinib [33,34]. Indeed, the SELECT trial indicated that the adjuvant erlotinib might extend DFS in patients with *EGFR*-mutant stage IA–IIIA NSCLC, compared to the historical controls. However, subsequent randomized phase 2 and phase 3 clinical trials have yielded conflicting results on their superior effect on DFS compared to BSC or chemotherapy alone, and the OS rate was not improved in any of these studies [35,36,37,38,39,40,41,42] (Table 1). The randomized placebo-controlled RADIANT trial showed a non-significant increase in DFS for stage IB–IIIA with adjuvant erlotinib, with 37% of relapses involving the central nervous system (CNS) [39]. In the EVAN trial, adjuvant erlotinib improved the 2-year DFS rate over chemotherapy in stage IIIA patients with *EGFR* mutations [38]. In the ADJUVANT/CTONG1104 trial, gefitinib improved DFS, compared to chemotherapy, in the stage II–IIIA patients with *EGFR* mutations (hazard ratio—HR 0.60; 95% CI, 0.42 to 0.87; *p* = 0.005), although this benefit did not translate into improved OS. Despite these findings suggesting that patients with resected *EGFR*-mutant NSCLC could benefit from *EGFR*-TKIs, they did not enter clinical practice. In fact, clinical trials have been heterogeneous in terms of study design and population, adjuvant chemotherapy, years of adjuvant *EGFR*-TKI treatment, and results [43]. Furthermore, adverse events such as skin rash and gastrointestinal toxicity were considered to significantly affect the quality of life in a curative setting.

Oral irreversible, mutant-selective, CNS-active, third-generation *EGFR*-TKI osimertinib has significantly improved clinical outcomes in the metastatic setting [22,23]; thus, the phase 3 randomized, double-blind, placebo-controlled ADAURA trial also investigated its effect in the adjuvant setting in 682 patients with completely resected stage IB (≥4 cm)–IIIA (AJCC—American Joint Committee on Cancer—VII edition) NSCLC harboring *EGFR* exon 19 deletions or exon 21 L858R mutations who had radical tumor resection, with or without prior adjuvant chemotherapy [44]. In this trial, patients were stratified by the type of *EGFR* mutation (Ex19del/L858R), disease stage (IB/II/IIIA), and race (Asian/non-Asian), and they were randomly assigned 1:1 to receive either 3-year oral osimertinib 80 mg once daily or a placebo until disease recurrence or death. Baseline characteristics of the enrolled population were well balanced between the two groups and representative of the general population; 64% of patients were Asians and approximately 70% were female. The majority of patients had an adenocarcinoma histology and had undergone a lobectomy, and 60% had received adjuvant platinum-based chemotherapy. *EGFR* exon 19 deletions were predominant (55%) compared to exon 21 L858R (45%) [44].

The primary endpoint was met as the trial demonstrated a statistically significant and clinically meaningful improvement in DFS among patients with stage II–IIIA compared to the placebo with an 83% reduction in the risk of recurrence or death (HR 0.17; 99.06% CI, 0.11 to 0.26; *p* < 0.001), leading to the FDA approval of adjuvant osimertinib in 2020 [44]. The benefit was also observed in the overall stage IB–IIIA population, with an 80% reduction in the risk of recurrence or death (HR 0.20; 99.12% CI, 0.14 to 0.30; *p* < 0.001). The favorable benefit of osimertinib was observed in all the pre-specified subgroups, including different disease stages and irrespective of the use of adjuvant chemotherapy. Furthermore, osimertinib was associated with a reduced risk of loco-regional (7% vs. 18%) and distant (4% vs. 28%) recurrence and CNS relapse (1% vs. 10%). Most frequent adverse events included diarrhea, paronychia, and dry skin, and discontinuation owing to adverse events occurred in 11% vs. 3% in the osimertinib and placebo arms, respectively, confirming a maintained quality of life in this setting. Interstitial lung disease occurred in 3% of patients treated with osimertinib and were generally mild or moderate [44].

With a median follow-up duration of 59.9 and 56.2 months in the osimertinib and placebo groups, respectively, among patients at stage II–IIIA, the 5-year OS rate was 85% and 73%, respectively (HR, 0.49; 95.03% CI, 0.33 to 0.73; *p* < 0.001), and in the overall population (stage IB–IIIA), the 5-year OS was 88% vs. 78% (HR, 0.49; 95.03% CI, 0.34 to 0.70; *p* < 0.001), regardless of disease stage and adjuvant chemotherapy [29].

### 2.2. Neoadjuvant Targeted Treatments in EGFR-Mutant Setting

In the neoadjuvant setting, a single-arm phase 2 study evaluated neoadjuvant gefitinib among patients with *EGFR*-mutant, stages II to IIIA NSCLC, with encouraging results. In particular, the overall response rate (ORR), the primary endpoint, was 54.5%, major pathologic response (MPR) was 24.2%, and median DFS was 33.5 months. No grade 3 or 4 adverse events (AEs) were reported, and MPR was associated with improved survival [45].

Indeed, there is evidence supporting the use of MPR and pathologic complete response (pCR) as a surrogate for OS in neoadjuvant studies in resectable stage I–III NSCLC, with an independent prognostic role [46].

Erlotinib was evaluated in a phase-2 study of Chinese patients with stage IIIA *EGFR*-mutant NSCLC in comparison with chemotherapy. Erlotinib use led to a higher ORR (67% vs. 19%), pathologic response rate (67% vs. 38%), and OS (51.0 vs. 20.9 months) than cisplatin-based doublet chemotherapy [47].

Another randomized, multicentric, phase 2 trial, the EMERGING-CTONG 1103, compared neoadjuvant chemotherapy with erlotinib in patients with stage IIIA N2 *EGFR*-mutant NSCLC. Despite the fact that the primary endpoint of ORR was not met, median progression free survival (PFS) improvement was observed (21.5 vs. 11.4 months, respectively) but did not translate into an OS benefit [48,49].

Neoadjuvant osimertinib was evaluated in a small phase 2 study of 27 patients with stages I to IIIA *EGFR*-mutant NSCLC, showing 15% MPR, 48% partial response, and 44% lymph node downstaging [50].

Important results also came from the NEOS study, a multicenter single-arm open-label phase 2 trial, in which 40 Chinese patients diagnosed with *EGFR*-mutant NSCLC were enrolled to be treated with neoadjuvant osimertinib therapy for six weeks [51]. Among the 38 patients who completed osimertinib treatment, the primary end point of ORR was 71.1%, with a successful R0 resection in 93.8% of patients and MPR in 10.7%. Alongside the clinical benefit, neoadjuvant osimertinib has also shown a manageable safety profile, with only three adverse events of grade 3 and none of them leading to dose reduction or discontinuation [51].

Focusing on these results and following the strong evidence of increased benefit from combining an *EGFR*-TKI with chemotherapy in treatment-naïve advanced *EGFR*-mutant NSCLC [52], clinical trials have been designed to evaluate this strategy in the neoadjuvant setting as well.

In particular, the phase 3 randomized NeoADAURA trial, is evaluating the efficacy and safety of neoadjuvant osimertinib, as monotherapy or in combination with chemotherapy, vs. chemotherapy with placebo in patients with resectable stage II–IIIB (N2) *EGFR*-positive NSCLC [53]. The primary endpoint is the centrally assessed MPR at resection (defined as <10% residual viable cancer cells), after neoadjuvant osimertinib as monotherapy or in combination with chemotherapy compared with neoadjuvant chemotherapy with placebo [53]. Very recently, negative results from a small phase 2 multicenter trial (*n* = 27 patients) have been published, showing that 56-day neoadjuvant osimertinib treatment did not meet primary endpoint (MPR 14.8%) [54].

### 2.3. Targeted Treatments in EGFR-Mutant Locally Advanced Setting

In parallel with the other attempts to extend the use of *EGFR*-TKIs in the early-stage setting, the *EGFR*-TKI strategy has started to be explored in unresectable stage III *EGFR*-mutant NSCLC as well (Table 2).

Among the main reasons behind this attempt, apart from the preclinical hypothesis of a radiosensitizing effect of *EGFR*-TKIs, there is evidence to suggest that patients with locally advanced *EGFR*-mutant NSCLC have a better survival rate but inferior distant control, especially central nervous system (CNS) metastases vs. those with *EGFR* wild-type NSCLC [55].

The randomized phase II RECEL (NCT0174908) enrolled 41 unresectable *EGFR*-mutant stage III NSCLC patients, who were randomized to radiotherapy plus erlotinib for 2 years or cCRT. PFS, the primary endpoint, was significantly improved in the erlotinib compared with the cCT-RT arm (24.5 vs. 9 months, HR 0.104, 95% CI: 0.028–0.389, *p* < 0.001), with similar incidence of adverse events [56].

A similar strategy was explored in the single-arm phase II WJOG6911L study with gefitinib, showing a favorable response rate (81.5%) and 30% PFS rate at 2 years [57].

However, the above-mentioned studies are limited by small patient numbers and the use of early generation *EGFR*-TKIs, thus they do not support the superior efficacy or comparable safety of tested TKI regimens in locally advanced (LA)-NSCLC.

The randomized phase III LAURA trial was conducted to formally evaluate the safety and efficacy of osimertinib consolidation vs. the placebo after cCRT for unresectable *EGFR*-mutant NSCLC [31]. In this trial, 216 patients with no investigator-assessed disease progression after ≥2 cycles of either concurrent or sequential platinum-based CRT were randomized to receive osimertinib or placebo (2:1) until disease progression or unacceptable toxicities. PFS, the primary endpoint of the study, was significantly prolonged with osimertinib (median PFS 39.1 vs. 5.6 months with placebo; HR 0.16, 95% CI 0.10–0.24; *p* < 0.001), with 74% vs. 22% of patients being alive and progression free at 12 months. The incidence of new brain lesions was also lower in patients who received osimertinib (8%) compared to the placebo (29%) [31].

OS data were still immature at the time of the first analysis presented but, given the striking surrogate results of PFS and CNS disease control, this approach is expected to become the new standard of care in the near future for patients with unresectable locally advanced *EGFR*-positive NSCLC.

## 3. *ALK*-Rearranged Setting

### 3.1. Adjuvant Targeted Treatments in ALK-Rearranged Setting

Approximately 4–5% of NSCLCs harbor *ALK* alterations, which are associated with younger age, never smokers, and generally advanced disease, with a high risk of brain metastases [58]. In the adjuvant setting, platinum-based chemotherapy has been the mainstay, with only a modest improvement in disease outcomes, alongside significant toxicity. Furthermore, in a retrospective cohort study that included 309 patients with surgically resected stage IA lung adenocarcinoma, patients with an *ALK*-positive tumor had a significantly lower 5-year DFS rate (62.4%) compared to those with *ALK*-negative tumors (86.5%; *p* = 0.038), underscoring that *ALK* rearrangements are independent risk factors for recurrence [59]. The advent of ICIs in the adjuvant setting has not led to a change in the treatment algorithm for *ALK*-positive NSCLCs, as there is no clinical evidence supporting a benefit of immunotherapy in NSCLC patients with oncogenic driver mutations.

In the phase 3 ALEX trial, first-line *ALK*-TKI alectinib significantly improved PFS and OS compared to crizotinib, with high efficacy in terms of CNS disease control [24,60]. Moreover, this agent was associated with a favorable safety profile [24]. Based on its efficacy in the advanced setting, alectinib was evaluated as part of postoperative treatment strategy.

The phase 3, open-label, randomized ALINA trial randomized 257 patients with completely resected stage IB (≥4 cm)-IIIA (AJCC VII edition) *ALK*-positive NSCLC to receive 2 years of adjuvant alectinib vs. four cycles of platinum-based chemotherapy [30]. In this trial, the baseline characteristics were well matched between the two groups. A majority of the patients were never smokers (64% vs. 55% in the alectinib and chemotherapy group, respectively), and they had undergone a lobectomy with a diagnosis of non-squamous histology. In the alectinib arm, approximately 54% of patients had stage IIIA disease, and 50% had pN2 disease, whereas in the chemotherapy group, 55% of patients had stage IIIA disease, with 52% having pN2 disease [30]. Notably, no patients had received neoadjuvant therapy nor postoperative radiotherapy. Alectinib led to a 76% lower risk of disease recurrence or death compared to chemotherapy in patients with stage IB (≥4 cm)–IIIA (HR 0.24; 95% CI, 0.13 to 0.43; *p* < 0.001) NSCLC, a benefit that was consistent throughout all subgroups of patients [30]. At an exploratory analysis, CNS disease recurrence was also reduced by adjuvant alectinib (HR, 0.22; 95% CI, 0.08 to 0.58), although it was reported as the first site of progression, with a 78% lower risk in alectinib arm [30]. Overall survival data are still immature, and a longer follow-up will be crucial to determine the impact of alectinib on OS. After a median follow-up for safety of 23.9 and 2.1 months for alectinib and chemotherapy, respectively, the adverse events were manageable and consistent with data from alectinib in the metastatic setting, including an increase in creatinine and aspartate- and alanine-aminotransferase. Furthermore, adverse events leading to permanent treatment discontinuation were more frequent with chemotherapy (5.5% vs. 12.5%) [30].

On the basis of these results, FDA and EMA approved alectinib as an adjuvant treatment for completely resected, stage IB–IIIA, *ALK*-positive NSCLC.

### 3.2. Neoadjuvant Targeted Treatments in ALK-Rearranged Setting

Moving to the neoadjuvant setting, Zhang et al. evaluated neoadjuvant crizotinib as feasible and well tolerated in 11 patients with resectable *ALK*-positive NSCLC, obtaining a partial response in 10 out of 11 patients and one stable disease. R0 resection was performed in ten patients, two of them achieving pCR [61].

Of note, alectinib was found to have a better outcome than crizotinib (pCR: 37.5% vs. 15.4%) in a retrospective study of patients with stage III *ALK*-positive NSCLC who had received surgery after induction therapy of alectinib (*n =* 16) or crizotinib (*n =* 13) [62].

Based on these data, multiple ongoing clinical trials are investigating the efficacy and safety of newer-generation *ALK* inhibitors in the neoadjuvant setting (Table 3).

Indeed, data from a preliminary analysis among the *ALK*-positive cohort of NAUTIKA1 have already shown the feasible profile of alectinib for neoadjuvant treatment as follows: All patients underwent surgery during the defined protocol window and had a complete resection (R0 resection rate: 100%) without delays or major complications [63].

Of note, in the ALNEO Trial, patients with potentially resectable stage III *ALK*-positive NSCLC (any T with N2, T4N0-1) were randomized to receive oral alectinib 600 mg twice daily for two cycles of 4 weeks each (8 weeks in total) during the neoadjuvant phase, followed by adjuvant alectinib for 24 cycles (96 weeks) after radical surgery [64].

### 3.3. Targeted Treatments in ALK-Rearranged Locally Advanced Setting

Similarly to the *EGFR*-mutant setting, targeted approaches are also expected to improve survival outcomes in the *ALK*-positive unresectable locally advanced disease.

Signals come from a retrospective study of 212 patients with unresectable stage II/III NSCLC who had received definitive chemoradiation and consolidation treatments. In this study, 53% of those with available genetic testing (89.9%) had driver gene alterations (58.5% *EGFR* mutations, 22.5% *KRAS* mutations, 6.3% *ALK* mutations, 12.7% others), while 47% were identified as wild type. Up to 72 patients (64.9%) among those with driver gene mutations received consolidation therapy, including targeted therapies (osimertinib and alectinib) along with immunotherapies [65]. Of note, targeted consolidation therapy considerably prolonged the PFS, though it did not significantly increase OS [65].

The phase II RTOG 1306 (NCT01822496) trial randomizing *ALK*-positive patients to receive either crizotinib induction for 3 months followed by chemotherapy plus concurrent radiotherapy (60 Gy/30 F) or cCRT alone was terminated due to the poor accrual rate.

## 4. Ongoing Clinical Trials for Actionable Oncogenic Drivers in the Non-Metastatic Setting

Several clinical trials are ongoing to address the clinical need for targeted treatment strategies in the early setting of resectable and unresectable NSCLCs, including other oncogenic drivers beyond *EGFR* and *ALK* (Table 2, Table 3 and Table 4). However, to date, no definitive clinical data have been published so far.

In patients with *EGFR*-mutant NSCLC, ongoing clinical trials are investigating the efficacy of adjuvant osimertinib for patients with stage IA2–IA3 disease (phase 3 ADAURA2; NCT05120349) and whether extending therapy beyond three years to five years might be beneficial (phase 2 single-group TARGET; NCT05526755). Notably, the TARGET study will also address the efficacy of adjuvant osimertinib for patients with NSCLC harboring *EGFR* uncommon mutations, addressing a clinically unmet need [66].

For stage IB–IIIA *ALK*-positive patients, crizotinib (ALCHEMIST trial, NCT02201992) and ensartinib (NCT05341583) are under evaluation in phase 3 clinical trials.

Ultimately, earlier molecular analysis and treatment are crucial to improving the chances of curative treatments, and further clinical research is warranted to establish novel strategies and their most effective duration for oncogene-addicted resectable NSCLC [67].

**Table 2:** Ongoing clinical trials with targeted therapies in the adjuvant setting.

Trial ID	Phase	Stage	Mutation	Treatment	Duration of Treatment, Years	Primary End Point	Status
NCT05120349 (ADAURA2)	III	IA2-IA3	*EGFR* Ex19del or L858R	osimertinib placebo	3 years	DFS in high risk	Recruiting
NCT06323148 (ETOP-1022)	III	II–IIIA (N1-N2)	*EGFR* Ex19del or L858R	osimertinib guided by ctDNA-MRD	3 years	3-year DFS rate	Not yet recruiting
NCT06080776	III	II, IIIA, IIIB (T3N2M0)	*EGFR* Ex19del or L858R	SH-1028 (oritinib) vs. placebo	3 years	DFS	Recruiting
NCT06041776	III	IB–IIIB (T3N2M0)	*EGFR* Ex19del or L858R	befotertinib + icotinib placebo vs. icotinib + befotertinib placebo	3 years	DFS in stage II–IIIB	Recruiting
NCT04687241	III	II–IIIB	*EGFR* Ex19del or L858R	almonertinib vs. placebo	3 years	DFS	Active, not recruiting
NCT05526755 (TARGET)	II	II–IIIB	*EGFR* Ex19del or L858R; *EGFR* uncommon (Ex20ins. excluded)	osimertinib	5 years	DFS	Recruiting
NCT05686434 (OSTAR)	II	I with high-risk factors (solid and/or micropapillary component ≥ 10%, and/or airway spread)	*EGFR* Ex19del or L858R	osimertinib	3 years	3-year DFS rate	Recruiting
NCT05514314	II	IA–IB with high risk features	*EGFR* Ex19del or L858R	icotinib	2 years	RFS	Not yet recruiting
NCT02264210	II	IB	*EGFR* Ex19del or L858R	icotinib or observation	12 months	DFS	Recruiting
NCT05536505	II	IB–IIIB (MRD positive)	*EGFR* Ex19del, L861Q, G719X, L858R	icotinib or osimertinib (if T790M positive)	Until MRD negative	DFS, 3-year DFS rate	Recruiting
NCT06227897 (ARESA)	II	IB (≥4cm), II and IIIA	*EGFR* Ex19del or L858R	aumolertinib	3 years	3-year DFS rate	Not yet recruiting
NCT04922138 (APPOINT)	II	I with solid, micropapillary, and/or complex gland components ≥ 10%	*EGFR* Ex19del or L858R	aumolertinib	3 years	2-year DFS rate	Recruiting
NCT05445310	II	IA with High Risk Factors and Stage IB	*EGFR* Ex19del or L858R and uncommon mutations (S768I, G719X, L861Q, T790M)	furmonertinib	3 years	3-year DFS rate	Recruiting
NCT05165355 (ATHEM)	II	IB–IIA with high-risk pathological subtype	*EGFR* Ex19del or L858R	furmonertinib	3 years	2-year DFS rate	Recruiting
NCT06192849	II	IB–IIIA	*EGFR* Exon 20 insertion mutations	furmonertinib	3 years	DFS	Recruiting
NCT05546866	II	IB–IIIB	Uncommon *EGFR* mutations (G719X/L861Q/S768I/de novo T790M)	osimertinib	3 years	3-year DFS rate	Recruiting
NCT02201992 (ALCHEMIST)	III	IB–IIIA	*ALK* fusions	crizotinib *versus* observation	2 years	OS	Recruiting
NCT05341583	III	II, IIIA or IIIB (T3N2M0)	*ALK* fusions	ensartinib *versus* placebo	2 years	DFS	Recruiting
NCT05241028	II	IB–IIIA	*ALK* fusions	ensartinib	3 years	3-year DFS rate	Recruiting
NCT05186506	II	IIA–IIIA	*ALK* fusions	ensatinib or platinum-based chemotherapy	2 years	DFS	Not yet recruiting
NCT04819100 (LIBRETTO-432)	III	IB–IIIA	RET fusion-positive	selpercatinib	3 years	EFS in stage II–IIIA RET fusion-positive NSCLC	Recruiting

Abbreviations: *ALK*: anaplastic lymphoma kinase; ctDNA: circulating tumor DNA; DFS: disease-free survival; EFS: event-free survival; EGFR: Epidermal Growth Factor Receptor; HR: hazard ratio; MRD: minimal residual disease; OS, overall survival; RET: rearranged during transfection; RFS: relapse-free survival; TKIs: tyrosine kinase inhibitors.

Neoadjuvant targeted treatments are also investigated in other actionable gene mutations, either in basket studies or in dedicated trials. Ongoing trials with targeted treatments in the neoadjuvant setting are summarized in Table 3.

**Table 3:** Ongoing clinical trials with targeted therapies in the neoadjuvant setting.

Trial ID	Phase	Stage	Mutation	Treatment	Duration of Neoadjuvant Treatment	Adjuvant Treatment	Primary End Point	Status
NCT04351555 (NeoADAURA)	III	II–IIIB N2	*EGFR* Ex19del or L858R (either alone or in combination with other *EGFR* mutations (ie, T790M, G719X, Exon20 insertions, S7681 and L861Q)	osimertinib + PBC vs. placebo + PBC or osimertinib monotherapy	3 cycles	No	MPR	Recruiting
NCT03203590	III	II–IIIA	*EGFR* Ex19del or L858R	PBC vs. gefitinib	2 cycles 8 weeks	No	2-year DFS	Not yet recruiting
NCT05011487 (NOCE01)	II	N2 positive non-squamous NSCLC with resectable (Stage IIIA or T3-4N2 IIIB) disease	*EGFR* Ex19del or L858R (either alone or in combination with other *EGFR* mutations, ie, T790M, G719X, Exon20 insertions, S7681 and L861Q)	osimertinib + PBC	60 days (2 cycles)	No	Complete lymph node clearance rate (ypN0)	Recruiting
NCT06268210	II	IB–IIIB	*EGFR* Ex19del or L858R	lazertinib or lazertinib, pemetrexed, carboplatin	3 years 3 cycles	Lazertinib up to 3 years (both before and after surgery)	Primary pathological response	Not yet recruiting
NCT05469022 (NeolazBAL)	II	I–IIIB IVA (single metastasis)	*EGFR* Ex19del or L858R alone or concurrent rare *EGFR* gene mutations (T790M, G719X, exon 20 insertion, S768I)	lazertinib	9 weeks	3 years after surgery (stage >2)	ORR at 9 weeks	Recruiting
NCT05104788	II	IIA–IIIB	*EGFR* Ex19del or L858R	icotinib + platinum-based chemotherapy	2 cycles	No	MPR	Recruiting
NCT03749213	II	IIIA (N2)	*EGFR* Ex19del or L858R	icotinib	8 weeks	2 years	ORR	Recruiting
NCT05132985	II	II–IIIB (N2)	*EGFR* Ex19del or L858R	icotinib + platinum-based chemotherapy	2 cycles	2 cycles of PBC on day 1 with intercalated icotinib (D8-15) every 3 weeks, and continued icotinib for 2 years	MPR	Not yet recruiting
NCT05987826	II	II–IIIB (cT3N2)	*EGFR* Ex19del or L858R with or without other *EGFR* mutations	furmonertinib	8 weeks	No	ORR at 8 weeks	Not yet recruiting
NCT05430802 (FORESEE)	II	IIIA/IIIB	*EGFR* Ex19del or L858R with or without other *EGFR* mutations	furmonertinib + platinum-based chemotherapy	3 cycles	No	ORR	Recruiting
NCT04685070	II	III	*EGFR* Ex19del or L858R	almonertinib	2–4 cycles (4 weeks per cycle)	Up to 48 weeks (including neoadjuvant phase)	ORR	Active, not recruiting
NCT04455594 (ANSWER)	II	IIIA (N2)	*EGFR* Ex19del or L858R with or without other *EGFR* mutations	almonertinib Investigator-choice therapy (erlotinib or chemotherapy)	3 cycles	No	ORR	Not yet recruiting
NCT05015010 (ALNEO)	II	III (any T with N2, T4N0-1)	*ALK* fusion	alectinib	8 weeks	96 weeks	MPR	Recruiting
NCT04302025 (NAUTIKA-1)	II	IB, IIA, IIB, IIIA, or selected IIIB (T3N2 only)	*ALK* fusion, *ROS1* fusion, *NTRK1*/*2*/*3* fusion; *BRAF* V600 mutation (enrollment closed); *RET* fusion (enrollment closed), *KRAS* G12C	alectinib entrectinib pralsetinib divarasib	8 weeks	2 years of targeted therapies	MPR	Recruiting
NCT06282536 (LungMate-018)	II	III–IVA	*ALK* fusion	iruplinalkib	4 cycles	2 years	ORR	Not yet recruiting
NCT05380024 (NEOEAST)	II	IIA–IIIB	*ALK* fusion	ensartinib	8 weeks	4 cycles adjuvant PBC followed by 2 years targeted therapy	MPR	recruiting
NCT05472623 (Neo-KAN)	II	IB–IIIA	KRAS G12C	adagrasib or adagrasib/nivolumab	6 weeks	PBC ± radiotherapy	pCR	Recruiting
NCT05118854	II	IIA–IIIB (T3-4N2)	*KRAS* G12C	sotorasib + PBC	4 cycles	No	MPR	Recruiting
NCT06054191	II	IB–IIIA and selected IIIB (T3N2, T4N2)	*BRAF* V600E *MET* ex14 skipping	dabrafenib and trametinib (*BRAF* cohort) capmatinib (*MET* cohort)	8 weeks	4 cycles of adjuvant PBC followed by 2 years targeted therapy	pCR	Not yet recruiting
NCT04712877	Observational	IA2-III	10 oncogenic drivers detected by ctDNA (*EGFR*, *BRAF* V600E, *MET* exon 14, *HER2*, *ALK*, *RET*, *NTRK*, *ROS1*, amplification of *MET* and *HER2*)	Clinical trial of neoadjuvant targeted therapy	NA	NA	Proportion of Patients who Possess Actionable Oncogenic Drivers	Recruiting

Abbreviations: *ALK*: anaplastic lymphoma kinase; *BRAF*: v-raf murine sarcoma viral oncogene homolog B1; ctDNA: circulating tumor DNA; *EGFR*: Epidermal Growth Factor Receptor; *HER2*: human epidermal growth factor receptor 2; HR: hazard ratio; *KRAS*: Kirsten rat sarcoma virus; MPR: major pathological response; NSCLC: non-small cell lung cancer; NTRK: Neurotrophic tyrosine receptor kinase; ORR: objective response rates; pCR: pathological complete response; PBC: Platinum-based chemotherapy; RET: rearranged during transfection; *ROS1*: c-ros oncogene 1.

To date, a few trials are ongoing evaluating the role of TKIs in the consolidation setting in *EGFR*, *ALK*, and *ROS1* positive patients (Table 4).

**Table 4:** Ongoing phase 2–3 trials in driver-mutant unresectable locally advanced NSCLC.

Trial ID	Phase	Stage	Driver Gene Alteration	Treatment	TKI Duration	Primary End Point
**NCT05351320**	2	Unresectable locally advanced	*ALK* *ROS1*	iruplinalkib WX-0593 with cCRT	Until PD or unacceptable toxicity	G ≥ 3 pneumonitis
**NCT05718297**	2	Unresectable locally advanced	*ALK *	brigatinib vs. durvalumab vs. observation after CRT	Until PD or unacceptable toxicity	PFS
**NCT04636593**	2	Unresectable locally advanced	*EGFR *	almonertinib with CRT	Until PD or unacceptable toxicity	G ≥ 3 pneumonitis
**NCT05338619 (PLATINUM) **	2	Unresectable locally advanced	*EGFR *	lazertinib after cCRT	Until PD or unacceptable toxicity	PFS

Abbreviations: TKI: tyrosine kinase inhibitor; *ALK*: anaplastic lymphoma kinase; *ROS1*: c-ros oncogene; *EGFR*: Epidermal Growth Factor Receptor; cCRT: concurrent chemo-radiation; CRT: chemo-radiation; PD: progressive disease; G: grade; PFS: progression free survival.

## 5. The Role of Immunotherapy

Immune checkpoint inhibitors have modified the standard of care in the metastatic and, more recently, in the early-stage setting. However, growing evidence is building up from the metastatic setting on the lack of efficacy of ICIs in patients with most actionable driver gene alterations, especially when administered as monotherapy [68,69].

ICIs have been recently introduced in the treatment armamentarium, although the selection of patients still requires a finer stratification. In October 2022, based on the results of the phase 3 trial Impower-010, the Food and Drug Administration (FDA) approved adjuvant anti-PD-L1 atezolizumab for patients with stage II–IIIA (according to the AJCC VII edition) NSCLC and a PD-L1 expression >1%, after surgery and platinum-based chemotherapy [70]. Given the significant improvement in the rate of disease-free survival (DFS) in this trial [71] and a hazard ratio (HR) for patients with PD-L1 ≥ 50% of 0.43 (95% CI [0.27, 0.68]) [71], confirmed at a 5-year-follow-up (median DFS NE—not estimated—vs. 41.1 months for BSC (HR, 0.48; 95% CI, 0.32–0.72) [72], alongside a positive overall survival (OS) trend at a median follow up of 45.3 months, for patients with stage II–III and a PD-L1 ≥50% (median OS NE vs. 87.1 months, HR, 0.47; 95% CI, 0.28–0.77) [73], these led to the EMA approval of adjuvant atezolizumab only for patients with a PD-L1 ≥ 50% [72]. Conversely, in the phase 3 KEYNOTE-091/PEARLS study, pembrolizumab was not able to significantly improve DFS in the PD-L1 ≥ 50% population (median not reached in either arm; HR 0.82) [74]. Furthermore, in a recent update on the phase 3 ADJUVANT BR.31 trial, adjuvant durvalumab failed to achieve a statistically significant improvement in DFS compared to placebo in patients with resected stage IB–IIIA *EGFR* and *ALK* wild-type NSCLC and with a PD-L1 expression ≥25% [75].

In this evolving therapeutic landscape, the population of patients harboring genomic alterations, including but not limited to, *EGFR* mutations and *ALK* rearrangements represents a niche with potentially worse prognosis, in which the use of ICIs in this subgroup of patients is not endorsed based on retrospective data of reduced efficacy in patients with oncogene-addicted NSCLC in the metastatic setting, with the exception of *KRAS*-mutant NSCLC.

As a biological background, it has been demonstrated that *ALK* and *EGFR* oncoproteins and downstream signaling are involved in the mechanism of PD-L1 upregulation and influence multiple intrinsic pathways leading to cancer cell immune evasion thus representing an intrinsic mechanism of resistance to immunotherapy treatments [76,77].

Pivotal adjuvant immunotherapy trials (namely IMpower010 and PEARLS) were initially designed prior to such evidence and did not require a mandatory test for any driver mutation, such that patients with known *EGFR* or *ALK* alterations were allowed to be included [71,74]. Hence, partial information is available in these selected populations and, as a direct consequence, controversial data have been obtained.

Indeed, 43 patients with known *EGFR* mutations and 23 patients with known *ALK* rearrangements were included in the stage II–IIIA PD-L1 ≥ 1% population in the IMpower010 trial (n= 185 and *n =* 199 not tested patients, respectively). No DFS or OS benefit was observed with adjuvant atezolizumab compared to the best supportive care in these mutated subgroups, even when looking only at the PD-L1 ≥5 0% population [71,73].

Conversely, DFS benefit with pembrolizumab vs. placebo was confirmed in the 73 patients (6%) with known *EGFR* mutations enrolled in the PEARLS trial (*n =* 370 patients with unknown *EGFR* status), whereas no formal subgroup analysis was conducted in the 14 (1%) patients with *ALK* rearrangement [74].

Among the pivotal trials in the perioperative setting, the most recently designed required testing for *EGFR* and *ALK* are able to exclude positive cases from enrolment [16]. *EGFR*/*ALK* molecular status was not mandatory in the KEYNOTE-671, where no formal subgroup analysis was conducted in the small subgroup of patients with known *EGFR* (*n* = 33, 4%) and *ALK* (*n =* 21, 2.6%) gene alterations, and in the AEGEAN trial, where the known 74 *EGFR*/*ALK*-positive patients were excluded from the efficacy analysis [13,14].

Following these results, adjuvant atezolizumab in resected stage II–III PD-L1 high patients has been approved only in *EGFR*/*ALK* wild-type population, whereas adjuvant and perioperative pembrolizumab regimens have been approved regardless of *EGFR*/*ALK* molecular status [78,79].

Similar considerations apply to unresectable locally advanced disease. The addition of durvalumab has improved overall survival at 5 years to 42.9% (vs. 33.4% with placebo), with one-third of patients being progression-free at 5 years in the pivotal phase 3 randomized PACIFIC trial. However, the EMA limited the indication for the consolidation treatment with durvalumab to those patients with PD-L1 expression ≥ 1%, following a subgroup analysis that was not prespecified suggesting a lack of benefit in this subgroup [80]. Patients with known *EGFR* (*n =* 35) and *ALK* (*n =* 8) positive NSCLCs were included in the PACIFIC trial, and a post hoc subgroup analysis of the *EGFR*-positive population suggested the absence of benefit in terms of PFS and OS with the use of durvalumab in addition to concurrent chemo-radiation (cCRT) [81]. With regards to *ALK*-rearranged patients, the very small number of patients enrolled limits the possibility to try to form any conclusion on the efficacy of durvalumab consolidation in this subgroup [82].

Similarly, PFS was numerically shorter (median 11.1 months) among patients with known *EGFR* mutation (*n =* 46, 7.9%) in the real-world phase 3 PACIFIC-R trial [83].

In a small multi-institutional retrospective analysis of 37 patients with unresectable stage III *EGFR*-mutated NSCLC who received concurrent CRT, 13 initiated durvalumab within a median of 20 days after cCRT completion [84]. Of them, only two patients completed 12 months of treatment, with five patients discontinuing durvalumab due to disease progression and five due to immune-related adverse events. Of 24 patients who completed cCRT and did not receive durvalumab consolidation treatment, 16 completed CRT alone and 8 completed cCRT with the induction or consolidation of *EGFR*-TKI. No significant PFS benefit was demonstrated between those with or without duvalumab consolidation treatment. Of note, despite small numbers, the cCRT with *EGFR*-TKI strategy was associated with longer median PFS (26.1 months) compared to cCRT alone or with durvalumab (*p* = 0.023) [84]. Another larger multi-institutional retrospective study in 136 stage III *EGFR* NSCLC patients showed a significantly longer 24-month PFS rate (86%) in patients treated with osimertinib consolidation treatment after concurrent chemo-radiation compared to those treated with durvalumab (30%) or in the observation cohorts (27%), with no difference in PFS between the durvalumab and the observation cohorts [85].

Despite this evidence, the durvalumab consolidation regimen is also formally approved in the treatment of locally advanced unresectable *EGFR*-mutated NSCLC. However, an ESMO expert consensus has clearly stated that the use of consolidated ICI therapy after curative-intent chemoradiotherapy is not recommended in *EGFR*-positive disease [86].

Overall, the results obtained with targeted treatments in the early stage and locally advanced setting support the absence of any role for immunotherapy in the adjuvant and neoadjuvant perioperative setting, at least for *EGFR-* and *ALK*-positive disease. In unresectable locally advanced disease, available data from LAURA trial allow to formally exclude immunotherapy use only in the *EGFR*-mutant population, as already suggested by ESMO expert consensus [31,86]. However, pending confirmatory trials, it is reasonable to extend this limitation to the *ALK*-rearranged population and potentially to other actionable gene alterations that are not known to be associated with the efficacy of immunotherapy in the metastatic setting, especially in never-smoker patients.

## 6. Discussion

Currently, surgery is the first treatment option for early-stage NSCLC (stages I—selected IIIB according to 8th TNM). Neoadjuvant or adjuvant systemic therapies are available for stages II to III and selected stage IB diseases [12].

In the adjuvant setting, a 1-year maintenance treatment with an immune checkpoint inhibitor, atezolizumab or pembrolizumab, has to be considered after adjuvant chemotherapy, according to PD-L1 expression and molecular status (atezolizumab ≥50% in resected stages II–III without *EGFR*/*ALK* gene alteration, pembrolizumab regardless of PD-L1 and molecular status) [73,74].

Regarding TKIs, adjuvant 3-year osimertinib and 2-year alectinib are approved in the treatment of resected stage IB–III *EGFR*-positive (without or following adjuvant chemotherapy) and resected stage II–III *ALK*-positive NSCLCs, respectively [29,30].

In the neoadjuvant setting, chemo-immunotherapy regimens represent the current new standard of care, with or without adjuvant immunotherapy, with approvals that do not exclude the use in patients with actionable gene alterations, including *EGFR* or *ALK*.

Similarly, consolidation treatment with durvalumab (with regional limitations according to PD-L1 status) after chemoradiation for unresectable locally advanced NSCLC has no regulatory limitations according to disease molecular status.

Despite the advances made in treatment effectiveness in these settings, metastatic recurrence is common and associated with poor survival [2], highlighting the need to define new therapeutic algorithms designed to be more effective on and tailored to the molecular profile of the disease.

In this view, a deeper knowledge of NSCLC molecular subtypes has led us to understand that tumors driven by a specific druggable genetic alteration often present with a particular phenotype and biological behavior, including a particularly high-risk of CNS metastases [87].

Cytotoxic chemotherapy plays a limited role in controlling brain metastases because of the drugs’ inability to cross the pharmacological sanctuary of the blood–brain barrier (BBB) and penetrate the CNS [88,89].

In the metastatic setting, the newer generation targeted systemic therapies, including those targeting *EGFR*, *ALK*, *ROS1*, *RET*, *BRAF*, *KRAS*, *MET*, and *NTRK*, have demonstrated greater CNS penetration with overall better intracranial ORR compared with standard chemotherapy, across treatment-naïve and pretreated patients [21]. Specifically, most TKIs were able to delay and prevent CNS progression in metastatic patients, with osimertinib and alectinib demonstrating their ability to also reduce the risk of CNS disease progression in the adjuvant setting [29,30].

Furthermore, data regarding immunotherapy are overall suggesting a lack of efficacy among patients with NSCLC harboring actionable driver genetic alterations (with only clear exception for *KRAS* mutations), especially in those who were never smokers, without any evidence of additional benefit from combining immunotherapy with targeted therapy [76,90,91].

A relevant issue when discussing neo-adjuvant treatments is the risk of treatment failure in non-responders, that might lead to missing curative surgery. This aspect is much more important when considering the formal possibility to treat driver-mutant resectable NSCLC with ICI-based regimens, that could impair the probability of tumor response and cancel the surgical option.

Of note, no broad molecular testing was required in the pivotal trials conducted with ICIs in the early stage and locally advanced setting, hence no information is available on the efficacy in mutant molecular subgroups other than *EGFR* or *ALK.* To bridge this gap, as in the metastatic setting, data from the real-world use of adjuvant and neoadjuvant ICI-based treatments would be of great value since they would be able to report data on surgical outcomes, recurrences, and survival, possibly according to molecular testing whenever performed during the disease course.

These data would be of relevance to ensure that patients receive the appropriate treatments for early stages and avoid toxicities with subsequent therapies.

Particular attention should be paid on safety when selecting therapies in the curative setting, as the treatment sequence may have an impact on toxicity. Indeed, an increased risk of hepatotoxicity was observed with the *ALK*-TKI crizotinib after chemo-immunotherapy [92], and also with the *KRAS* inhibitor sotorasib, when administered within 3 months of the last ICI administration [93]. Of note, osimertinib administration after chemo-immunotherapy was associated with the risk of pneumonitis [90,94].

Hence, the available evidence overall supports the extended use of TKIs in the non-metastatic setting. However, there is not a uniquely defined duration for TKI treatment, with a fixed duration selected in the adjuvant trials and a continuous duration in trials for unresectable locally advanced NSCLCs [67].

To date, data on the recurrence and outcomes after the permanent discontinuation of TKIs are not consistent, but signals from the decreasing DFS curve with osimertinib in the ADAURA trial after just 3 years suggest that a longer duration might further improve outcomes. Furthermore, the phase 2 TARGET trial was designed to compare a 5-year vs. 3-year duration of adjuvant osimertinib treatment [66]. However, given the heterogeneity of the study populations (according to stage, nodal status, and specific gene alteration subgroup), a proportion of patients might have already been cured with surgery, such that further prolonging the adjuvant treatment might result in dissipation of the beneficial effect. Furthermore, different trials used different diagnostic methods as they screened different patient populations and this may affect the interpretation of the results. As an example, in the ADAURA trial, PCR testing was used to screen for *EGFR* mutations. However, no information about the co-existing alterations (e.g., *TP53* mutations) which may cause intrinsic *EGFR*-TKI resistance would be available if NGS was not used. Therefore, it is important to mention that it has importance for the outcome as some patients may be cured with surgery only, while other patients may experience limited efficacy due to molecular heterogeneity.

To this extent, studies evaluating the pre- and post-surgical ctDNA and ctDNA monitoring during adjuvant TKI treatment would be helpful to better guide the most appropriate choice of treatment duration [95]. Such approaches could also be evaluated in neoadjuvant trials, in which it is worth acknowledging that, unlike the cytotoxicity of chemotherapy and enhanced immune surveillance obtained with ICIs, targeted therapies are cytostatic treatments. The difference in the mechanisms of action is the main reason for the lower pCR and MPR rates observed with neoadjuvant TKIs compared to those obtained with neoadjuvant chemo-immunotherapy (despite similar R0, downstaging, EFS or DFS outcomes) [16,28] thus potentially impacting the necessary duration of targeted therapies in the perioperative setting.

Overall, improving outcomes in the early stages of NSCLC with actionable gene alterations needs the implementation of upfront molecular testing in all stages, resulting in increased financial costs (testing, treatments, and resources), but also indirect costs for investing on the education of health professionals for an increasingly multidisciplinary approach. In parallel, it may also have impact on the social aspects and global health (potentially more cured patients staying within the labor market but with the need for longer follow-ups).

In conclusion, the overall results obtained with targeted treatments are modifying the treatment approaches of driver-mutant NSCLC in the early stages and in the locally advanced setting [96]. The increasingly and recommended widespread use of comprehensive genomic profile since the early stages will allow for a better characterization of the molecular subgroups and their response to treatments. Molecular-based dedicated trials are still ongoing; however, targeted therapies, apart from the already available adjuvant osimertinib or alectinib, are expected to further become an essential part of the treatment approaches deposing ICI use in most non-metastatic NSCLC harboring actionable gene mutations.

## Figures and Tables

**Table 1:** Clinical trials with available data with first-generation *EGFR*-TKIs in the adjuvant setting.

Trial (Phase)	Stage (AJCC Edition)	Treatment	DFS	OS
**BR19** **(III)**	IB–IIIA (6th)	gefitinib × 2 years vs. placebo (after adjuvant chemotherapy)	No difference (HR 1.22, 95% CI 0.93–1.61, *p* = 0.15)	No difference (HR 1.24, 95% CI 0.94–1.64, *p* = 0.14)
**ADJUVANT-CTONG1104** **(III)**	II–IIIA (7th)	gefitinib × 2 years vs. adjuvant chemotherapy	30.8 vs. 19.8 months (HR 0.56, 95% CI 0.40–0.79, *p* = 0.001)	75.5 vs. 62.8 months (HR 0.92, 95% CI 0.62–1.36, *p* = 0.674)
**IMPACT** **(III)**	II–III (7th)	gefitinib × 2 years vs. adjuvant chemotherapy	35.9 vs. 25.1 months (HR 0.92, 95% CI 0.67–1.28, *p* = 0.63)	No difference (HR 1.03, 95% CI 0.65–1.65, *p* = 0.89)
**RADIANT** **(III)**	IB–IIA (6th)	erlotinib × 2 years vs. placebo (after adjuvant chemotherapy)	50.5 vs. 48.2 months (HR 0.90, 95% CI 0.74–1.10, *p* = 0.324)	Not reached (HR 1.13, 95% CI 0.88–1.45, *p* = 0.335)
**SELECT** **(II)**	IA–IIIA (7th)	erlotinib × 2 years (after adjuvant chemotherapy)	Not reached (5-year DFS rate 56%)	Not reached (5-year OS rate 86%)
**EVAN** **(II)**	IIIA (7th)	erlotinib × 2 years vs. adjuvant chemotherapy	42.4 vs. 21.0 months (HR 0.27, 95% CI 0.14–0.53, *p* < 0.0001)	84.2 vs. 61.1 months (HR 0.32, 95% CI 0.15–0.67)
**EVIDENCE** **(III)**	II–IIIA (7th)	icotinib × 2 years vs. adjuvant chemotherapy	47.0 vs. 22.1 months (HR 0.36, 95% CI 0.24–0.55, *p* < 0.0001)	Not reached (HR 0.91, 95% CI 0.42–1.94)

Abbreviations: AJCC: American Joint Committee on Cancer; CI: confidence interval; DFS: disease-free survival; *EGFR*: Epidermal Growth Factor Receptor; HR: hazard ratio; OS, overall survival; TKIs: tyrosine kinase inhibitors.
